# Changes in diagnosis and operative treatment of insulinoma over two decades

**DOI:** 10.1007/s00423-023-02974-6

**Published:** 2023-06-29

**Authors:** D. Wiese, F. G. Humburg, P. H. Kann, A. Rinke, M. Luster, A. Mahnken, D. K. Bartsch

**Affiliations:** 1 Department of Visceral, Thoracic and Vascular Surgery, Philipps University, Marburg, Germany; 2 Department of Internal Medicine, Center for Endocrinology, Diabetology & Osteology, Philipps University, Marburg, Germany; 3 Division of Gastroenterology and Endocrinology, Department of Internal Medicine, Philipps University, Marburg, Germany; 4 Department of Nuclear Medicine, Philipps University, Marburg, Germany; 5 Department of Diagnostic and Interventional Radiology, Philipps University, Marburg, Germany

**Keywords:** Insulinoma, Laparoscopic, Diagnosis, Neuroendocrine, Parenchyma-sparing, Long-term

## Abstract

**Purpose:**

Most insulinomas are small solitary, benign neoplasms. Imaging and surgical techniques improved over the last 20 years. Thus, the aim of the present study was to analyze changes in diagnosis and surgery of insulinoma patients in a referral center over two decades.

**Methods:**

Operated patients with a histologically proven insulinoma were retrieved from a prospective database. Clinico-pathological characteristics and outcomes were retrospectively analyzed with regard to the time periods 2000–2010 (group 1) and 2011–2020 (group 2).

**Results:**

Sixty-one of 202 operated patients with pNEN had an insulinoma, 37 (61%) in group 1 and 24 (39%) in group 2. Of those 61 insulinomas, 49 (80%) were sporadic benign, 8 (13%) benign MEN1-associated insulinomas, and 4 (7%) sporadic malignant insulinomas. In 35 of 37 (95%) patients of group 1 and all patients of group 2, the insulinoma was preoperatively identified by imaging. The most sensitive imaging modality was endoscopic ultrasound (EUS) with correctly diagnosed and localized insulinomas in 89% of patients in group 1 and 100% in group 2. In group 1, significantly less patients were operated via minimally invasive approach compared to group 2 (19% (7/37) vs. 50% (12/24), *p* = 0.022). Enucleation was the most frequently performed operation (31 of 61, 51%), followed by distal resection (15 of 61, 25%) without significant differences between groups 1 and 2. The rate of relevant postoperative complications was not different between groups 1 and 2 (24% vs. 21%, *p* = 0.99). Two patients with benign insulinoma (1 out of each group) experienced disease recurrence and underwent a second resection. After a median follow-up of 134 (1–249) months, however, all 57 (100%) patients with benign insulinoma and 3 out of 4 patients with malignant insulinoma had no evidence of disease.

**Conclusion:**

Insulinoma can be preoperatively localized in almost all patients, allowing for a minimally invasive, parenchyma-sparing resection in selected patients. The long-term cure rate is excellent.

## Introduction

Insulinoma is a rare functioning neuroendocrine pancreatic tumor (pNEN) with an annual incidence of approximately 1–3/1,000,000 [1]. It presents as a potentially life-threatening disease caused by an insulin excess. Insulinoma often challenges clinicians regarding clinical presentation as well as diagnostic and surgical procedures [2]. The primary tumors are often less than 1 cm in size, are considered benign in at least 90% of cases, and can occur multifocal in the setting of multiple endocrine neoplasia type 1 (MEN1) or (pro)insulinomatosis [1–3]. Diagnosis of organic hyperinsulinism is established by a supervised fasting test, but preoperative localization of the underlying insulinoma might still be difficult [2, 4, 5]. From the 1980s to the early 2000s, surgical excision of the insulinoma after bidigital exploration of the pancreas and intraoperative ultrasound (IOUS) without further preoperative investigations was the procedure of choice, when a diffuse metastatic disease was excluded [6]. In the last decade, however, imaging modalities such as magnetic resonance imaging (MRI), computed tomography (CT), and endoscopic ultrasonography (EUS) have improved and new techniques such as Ga-68-DOTATOC-PET/CT and GLP-1-receptor-PET/CT have been introduced [1]. Thus, more recent guidelines recommend conclusive preoperative localization to allow minimally invasive parenchyma-sparing resection, whenever technically feasible [2, 7]. Even though enucleation represents the favored treatment for pancreatic preservation, it is used at present in only about 60% of patients with sporadic benign insulinoma [8]. This might be associated with a potentially problematic tumor location very close to the main pancreatic duct, in which case enucleation has significant risk of postoperative pancreatic fistula (POPF) type C with an up to 5× higher reoperation rate [9]. Despite this potential risk, enucleation provides long-term cure and prevents the risk of exocrine and endocrine pancreatic insufficiency [9]. A recent systematic review supports a laparoscopic approach for insulinoma resection in terms of safety, possibly reduced length of hospital stay, and cure rates comparable to open surgery [10]. The aim of the present retrospective study was to analyze the changes in diagnosis and surgical treatment over the last two decades in a tertiary referral center.

## Material and methods

The surgical prospective database of the Marburg ENETS (European Neuroendocrine Tumor Society) center of excellence was searched for patients who underwent surgery for insulinoma between January 2000 and December 2020. The insulinoma diagnosis was based on symptoms of hypoglycemia, a documented positive supervised fasting test, and a positive immunostaining of the tumor for insulin [2]. Tumors were classified as either sporadic or MEN1-associated in case of a proven MEN1 gene mutation and/or a typical family history [11].

For all identified patients, the demographics, preoperative imaging, type of surgery, postoperative complications, and long-term cure rates were analyzed regarding the years 2000–2010 (group 1) and 2011–2020 (group 2). Data collection and analysis was approved by the local ethics committee. Some data of analyzed patients were already previously reported [12].

Preoperative imaging routinely included abdominal ultrasonography, multidetector computed tomography (CT), and/or magnetic resonance imaging (MRI) with gadolinium and endoscopic ultrasonography (EUS). In most cases, EUS was performed by the same experienced investigator (PHK), in some cases combined with EUS-guided fine needle aspiration cytology (FNAC). Only selected patients underwent additional Ga-68-DOTATOC PET/CT.

Patients were planned for a laparoscopic approach, if the insulinoma was visualized preoperatively without signs of metastases and not located deep in the pancreatic head. In every potentially benign case, parenchyma-sparing resection was the goal, formal pancreatic resections were indicated only in patients with malignant insulinoma. Patients were eligible for enucleation in the absence of metastases and/or vascular involvement and a distance of at least 2 mm from the main pancreatic duct. Since December 2013, the daVinci Surgical System (Intuitive Surgical, Sunnyvale, CA, USA) was available and since then used in all laparoscopic pancreatic operations. In every open and laparoscopic procedure, IOUS was used to confirm the location of the insulinoma and its relation to vessels and the main pancreatic duct. No standard defect coverage of the pancreas was performed. In case of proven malignancy, systematic lymphadenectomy was performed; in potentially benign insulinomas, an intraoperative lymph node sampling was only performed in case of macroscopically suspicious lymph nodes.

Postoperative complications were classified according to Clavien-Dindo [13]. Clinically relevant postoperative pancreatic fistula (POPF) types B and C were defined according to the International Study Group of Pancreatic Fistula [14]. Length of hospital stay was not evaluated, because several institutional changes of patient demission management over the years would have introduced a significant bias.

Tumors were classified according to the WHO classification 2010 and defined as malignant in the presence of lymphatic and/or distant metastases at initial diagnosis or during follow-up [15].

Disease-free survival was defined as the time period from surgery to recurrent disease. An annual clinical follow-up was performed in the hospital or by the personal physician. In case of clinical suspicion for recurrence, a laboratory and imaging workup was performed in these patients to verify the recurrence of organic hyperinsulinism and/or a newly visible tumor.

### Statistics

All statistical analyses were performed using SPSS™ version 22. Two-sided *P* values < 0.05 were considered significant. Results are presented as the mean ± SD for normally distributed variables and as median and range for non-normally distributed variables. Descriptive statistics were used to summarize patient characteristics. Continuous variables were compared using the Mann-Whitney *U* test whereas categorical data were compared using Fisher’s exact test.

## Results

Between January 2000 and December 2020, a total of 202 patients were operated for pNEN, of which 61 (30%) patients had an insulinoma. The proportion of insulinoma patients among all resected pNEN patients was 39% (37 of 95) in group 1 (2000–2010) and 22% (24 of 107) in group 2 (2011–2020), respectively. Gender distribution, body mass index, and height were comparable between groups 1 and 2. Median age at surgery was 42 (range 10–82) years in group 1 and 52 (range 25–76, *p* = 0.029) years in group 2 (Table 1). The vast majority of patients in both groups had sporadic benign insulinomas (84% group 1 and 75% group 2), followed by MEN1-associated benign insulinomas (13% and 12%), only 4 patients (6.6%) had malignant insulinomas (Table 1).

**Table 1:** Demographic data of insulinoma patients

Parameter	Total cohort(*n* = 61)	Group 1(*n* = 37)	Group 2(*n* = 24)	*P* value^#^
Age at first operation (in years)^c^	48 (10–82)	42 (10–82)	52 (25–76)	0.029^a^
Height (in cm)^c^	173 (141–194)	172 (141–189)	173 (154–194)	0.762^a^
Weight (in kg)^c^	81 (35–180)	80 (35–180)	83 (56–130)	0.861^a^
BMI (in kg/m^2^)^c^	26.1 (17.6–42.4)	26 (17.6–73)	27.7 (18.9–42.4)	0.679^a^
Sex (M:W)	23:38	13:24	10:14	0.787^b^
Diagnoses				
Sporadic benign insulinoma	49 (80.3%)	31 (83.8%)	18 (75.0%)	0.463^b^
MEN-1 associated insulinoma	8 (13.1%)	5 (13.5%)	3 (12.5%)	
Sporadic malignant insulinoma	4 (6.6%)	1 (2.7%)	3 (12.5%)	

#*P* value was calculated between groups 1 and 2

^a^Mann-Whitney *U* test

^b^Fisher’s exact test

^c^Median with range

*BMI* body mass index

The diagnosis of organic hyperinsulinism was established in 36 (97%) patients of group 1 and in 24 (100%) patients of group 2 by a supervised positive fasting test. Preoperative imaging visualized the insulinoma correctly in 94.6% (35 of 37) patients of group 1 and in all (24 of 24) patients of group 2. The most sensitive imaging procedure in both groups was EUS with a detection rate of 89% in group 1 and 100% in group 2 (*p* = 0.151). It is of note that EUS was the only positive imaging procedure in 13 patients. Modern MRI in group 2 was more sensitive than in group 1, although not statistically significant (86% vs. 57%, *p* = 0.111). Somatostatin (SMS) receptor scintigraphy was used in only 11 of 37 (30%) patients in group 1 and in 4 of 24 (17%) patients in group 2 and was positive in 4 and 2 patients respectively. No patient in group 1 received a Ga-68-DOTATOC-PET-CT versus 8 (33%) patients in group 2 with a positive detection in all 8 cases. FNAC was performed more frequently in group 2 (12 of 24, 50%) compared to group 1 (5 of 37, 11%, *p* = 0.003). While FNAC was only performed in select cases during the earlier time period (only in cases with external or marginally pathological fasting test), it was regularly performed in later cases, as long as the suspicious pancreatic tumor was reachable. FNAC confirmed the pNEN in 14 of 17 (82%) patients. GLP-1-receptor-PET/CT was not performed in any of our patients. Median number of applied preoperative imaging techniques was 3 (range 1–5) in group 1 and 3 (range 2–4) in group 2 (*p* = 0.283). Preoperative imaging data are summarized in Table 2.

**Table 2:** Preoperative and intraoperative imaging of insulinoma patients

Diagnostic procedures	Total cohort (*n* = 61)	Group 1 (*n*** = **37)	Group 2 (*n*= 24)	*P* value^#^
Positive supervised fasting test	60/61 (98.3%)	36/37 (97.3%)	24/24 (100%)	>0.999^b^
Preoperative positive imaging	59/61 (96.7%)	35/37 (94.6%)	24/24 (100%)	0.515^b^
MRI	27/36 (75.0%)	8/14 (57.1%)	19/22 (86.4%)	0.111^b^
CT	17/31 (54.8%)	12/21 (57.1%)	5/10 (50.0%)	1.000^b^
EUS	53/57 (93.0%)	31/35 (88.6%)	22/22 (100%)	0.151^b^
Somatostatin receptor scintigraphy	6/15 (40.0%)	4/11 (36.4%)	2/4 (50.0%)	1.000^b^
Ga-68 PET-CT	8/8 (100%)	n.p.	8/8 (100%)	
GLP1 PET/CT	n.p.	n.p.	n.p.	
Fine needle aspiration cytology	14/17 (82.4%)	4/5 (80.0%)	10/12 (83.3%)	1.000^b^
Median number of preoperative imaging procedures^c^	3 (1–5)	3 (1–5)	3 (2–4)	0.283^a^

#*P* value was calculated between groups 1 and 2

Percentages in brackets relate to true positive localization

*n.p.* not performed, *MRI* magnetic resonance imaging, *CT* computed tomography, *EUS* endosonography

^a^Mann-Whitney *U* test

^b^Fisher’s exact test

^c^Median with range

Fifty-seven patients had benign insulinomas (36 in group 1 and 21 in group 2) and 4 patients had malignant insulinomas (1 in group 1 and 3 in group 2). Benign insulinomas in group 1 were more often located in the pancreatic head (53%) compared to group 2 (33%), although not statistically significant (*p* = 0.179). Benign insulinomas in group 2 were significantly more often operated via a minimally invasive approach (52%), when compared to group 1 (19%, *p* = 0.017). When localized in the pancreatic head, only one insulinoma out of each group respectively was resected minimally invasive (Table 3). IOUS was performed in almost every patient in both groups and always detected the pNEN(s). About half of patients with benign insulinoma in both groups had enucleations (56% in group 1, 52% in group 2, *p* = 1.000), followed by distal pancreatic resections (22% in group 1 and 24% in group 2) and partial pancreaticoduodenectomy (17% in group 1, 10% in group 2; Table 3), respectively.

**Table 3:** Localization, procedures, and postoperative complications in benign insulinoma

	Total cohort (*n* = 57)	Group 1 (*n* = 36)	Group 2 (*n* = 21)	*P* value^#^
Localization inside pancreas				
Head	26 (45.6%)	19 (52.8%)	7 (33.3%)	0.179^b^
Body/tail	31 (54.4%)	17 (47.2%)	14 (66.7%)	
Surgical access				
Conventional	39 (68.4%)	29 (80.6%)	10 (47.6%)	**0.017**^**b**^
MIS	18 (31.6%)	7 (19.4%)	11 (52.4%)	
MIS, if localization in head	2/26 (7.7%)	1/19 (5.3%)	1/7 (14.3%)	0.474^b^
MIS, if localization in body/tail	16/31 (51.6%)	6/17 (35.3%)	10/14 (71.4%)	0.073^b^
Surgical procedures				
Enucleation	31 (54.4%)	20 (55.6%)	11 (52.4%)	1.000^b^
DPR	13 (22.8%)	8 (22.2%)	5 (23.8%)	1.000^b^
PPD	8 (14.0%)	6 (16.7%)	2 (9.5%)	0.679^b^
Enuc. + DPR additional	5 (8.8%)	2 (5.6%)	3 (14.3%)	0.346^b^
Intraoperative ultrasound	56 (98.2%)	35 (97.2%)	21 (100%)	1.000^b^
Complications				
Clavien-Dindo ≥ 3	14 (24.6%)	9 (25.0%)	5 (23.8%)	1.000^b^
POPF grade B POPF grade C	5 (8.8%) 2 (3.5%)	2 (5.6%) 1 (2.8%)	3 (14.3%) 1 (4.8%)	0.346^b^ 1.000^b^
Non-surgical complications (LAE etc.)	5 (8.8%)	4 (11.1%)	1 (4.8%)	0.642^b^
Reoperation due to bleeding	6 (10.5%)	4 (11.1%)	2 (9.5%)	1.000^b^
Intraabdominal abscess	9 (15.8%)	5 (13.9%)	4 (19.0%)	0.712^b^

#*P* value was calculated between groups 1 and 2

*MIS* minimally invasive surgery, *Enuc* enucleation, *DPR* distal pancreatic resection, *PPD* partial pancreaticoduodenectomy, *POPF* postoperative pancreatic fistula, *LAE* pulmonary embolism

^a^Mann-Whitney *U* test

^b^Fisher’s exact test

All patients with benign insulinoma in both groups showed a postoperative reactive hyperglycemia. Clinically relevant postoperative complications (Clavien-Dindo ≥ 3) occurred in 9 of 36 (25%) patients with benign insulinoma in group 1 and 5 of 21 (24%) patients in group 2 (*p* = 1) (Table 2). Clinically relevant postoperative complications were not significantly different between patients who underwent laparoscopic (17%) or open (28%, *p* = 0.511) pancreatic resections (Table 4). There was also no significant difference in overall complications of patients who underwent either open or laparoscopic enucleations (24% vs. 10%, *p* = 0.634) or open versus laparoscopic distal pancreatectomy (29% vs. 33%, *p* = 1, Table 4).

**Table 4:** Postoperative complications after MIS and conventional surgery in benign insulinoma

Parameter	MIS total cohort (*n* = 18)	Open total cohort (*n* = 39)	*P* value	MIS enuc. (*n* = 10)	Open enuc. (*n* = 21)	*P* value	MIS DPR (*n* = 6)	Open DPR (*n* = 7)	*P* value
Clavien-Dindo ≥ 3	3 (17%)	11 (28%)	0.511^b^	1 (10%)	5 (24%)	0.634^b^	2 (33%)	2 (29%)	1.000^b^
POPF									
Grade B	2 (11%)	3 (8%)	0.646	1 (10%)	2 (9%)	1.000	1 (17%)	0	0.462
Grade C	0	2 (5%)	1.000	0	1 (5%)	1.000	0	0	
Reoperation due to bleeding	0	6 (15%)	0.162^b^	0	4 (19%)	0.277	0	0	
Intraabdominal abscess	2 (11%)	7 (18%)	0.704	1 (10%)	4 (19%)	1.000^b^	1 (17%)	0	0.462^b^
Wound infection	0	1 (3%)		0	1 (5%)		0	0	
Non-surgical complications (LAE etc.)	1 (6%)	4 (10%)	1.000^b^	0	0		1 (17%)	2 (28%)	1.000^b^

*MIS* minimally invasive surgery, *DPR* distal pancreatic resection, *POPF* postoperative pancreatic fistula, *LAE* pulmonary embolism

^a^Mann-Whitney *U* test

^b^Fisher’s exact test

The 4 patients with malignant insulinomas were aged 45, 52, 52, and 64 at first operation, all 4 patients had local lymphatic metastases, and two patients also had hepatic metastases. Three patients underwent open oncological pancreatic resections with systematic lymphadenectomy, including two distal pancreatectomies and one partial pancreatoduodenectomy. The fourth patient underwent a laparoscopic distal pancreatectomy, also with systematic lymph node dissection. In 3 of 4 patients, a complete tumor resection was achieved, in one patient with multiple liver metastases not all metastases were resectable, but about 90% of the liver tumor burden was debulked.

Histopathological analysis of the 57 benign insulinomas revealed a median tumor size of 15 (range 5–32) mm without significant differences beween groups 1 and 2 (Table 5).

**Table 5:** Comparison of histopathology and outcome between benign insulinomas of group 1 (2000–2010) and group 2 (2011–2020)

Variables	MIC group 1 (*n* = 7)	MIC group 2 (*n* = 11)	*P* value	Open group 1 (*n* = 29)	Open group 2 (*n* = 10)	*P* value
Tumor size in mm^c^	15 (11–22)	15 (11–23)	0.492^a^	14 (5–32)	13 (7–30)	0.935^a^
Resection status*						
R0	3/4 (75%)	10 (91.%)	0.476^b^	15/18 (83%)	6/8 (75%)	0.628^b^
R1	1/4 (25%)	1 (9%)		3/18 (17%)	2/8 (25%)	
Ki67 index*						
G1	7 (100%)	9 (82%)	0.497^b^	17/23 (74%)	8 (80%)	1.000^b^
G2	0	2 (18%)		6/23 (26%)	2 (20%)	
Positive IHC for insulin*	7 (100%)	11 (100%)		27/27 (100%)	10 (100%)	
Number of resected lymph nodes^c^	0 (0–1)	0 (0–5)	0.804^a^	1 (0–20)	3 (0–13)	0.173^a^
Median follow-up^c^	140 (48–192)	52 (13–89)		190 (3–249)	50.5 (1–110)	
Disease status at evaluation point*						
NED	6 (86%)	11 (100%)	0.389^b^	21/24 (87%)	10 (100%)	0.539^b^
DURC	1 (14%)	0		3/24 (13%)	0	

^a^Mann-Whitney *U* test

^b^Fisher’s exact test

^c^Median with range (months)

*IHC* immunohistochemistry, *NED* no evidence of disease, *DURC* dead of unrelated causes

*Missing data

After enucleation, the R1 resection rate was not significantly different in both groups after a laparoscopic approach (1 in group 1, 1 in group 2) compared to an open approach (2 in group 1, 0 group 2). The majority of patients had G1 tumors, 24 of 30 (80%) in group 1 and 17 of 21 (81%) in group 2 (*p* = 1). The median number of resected lymph nodes in a laparoscopic approach was 0 (range 0–5) and 2 (range 0 to 20) after an open approach with no statistically significant differences between groups 1 and 2. None of the 4 patients with malignant insulinoma had a clinically relevant postoperative complication.

After an overall median follow-up of 180 months in group 1 and 52 months in group 2, 2 of 57 (3.5%) patients with benign insulinoma experienced disease recurrence. The first patient initially received an enucleation of an insulinoma in the pancreatic head in 2005 and showed new signs of hypoglycemia in 2016. A recurrent pNEN was diagnosed in the pancreatic head, consistent with a local recurrence after 11 years, which was resected via partial pancreatoduodenectomy. For 57 months after the second operation, there have so far been no new signs of recurrence in this patient.

The other patient experienced recurrence of organic hyperinsulinism 4 months after the initial operation (enucleation). In a new diagnostic workup, multiple new nodules throughout the pancreatic tail, consistent with very small new insulinomas, were detected. After reoperation (left-sided pancreatic resection), the final pathological report showed the very rare diagnosis of adult pro-insulinomatosis [16]. Further workup showed no signs of multiple endocrine neoplasia in this patient, who has been relapse-free since the second operation as well for 38 months.

Of the 4 patients with malignant insulinoma, the patient with residual liver metastases and a very aggressive tumor histology (pNET G3, Ki67 70%) experienced recurrence of hypoglycemic symptoms as early as 2 months postoperatively. Over the following 12 months, this patient received further multimodal treatment for the later recurrence of i.v. glucose dependence and tumor progress, consecutively: everolimus, carboplatin/etoposide, chemo-embolization, dacarbazine/capeticabine, peptide receptor radionuclide therapy (PRRT). The patient died 14 months postoperatively from general tumor progress with multifocal distant metastases (liver, bone).

The other three patients with malignant insulinoma showed no evidence of relapse 14, 83, and 149 months, respectively, after the operation.

## Discussion

While intraoperative manual exploration and intraoperative ultrasound were standard procedures to detect clinically and biochemically proven insulinomas in the pre-2010 era, virtually all insulinomas were adequately localized with preoperative imaging in the last decade. In our patient cohorts, EUS was proven to be the most sensitive and reliable method to detect and localize insulinomas as small as 5 mm. This rate is rather high compared to other series [8], which might be due to the fact that all EUS were performed by the same experienced endosonographer. For our insulinoma patients, functional imaging (SMS-scintigraphy, Ga-68-DOTATOC-PET/CT) was rarely applied, because the information-benefit in otherwise already localized benign insulinomas was not seen.

When comparing the two patient groups (2000–2010 and 2011–2020), minimally invasive surgery has been increasingly established for patients with tumors in the left pancreas in the more recent cohort and proven to be equally feasible and safe as an open approach. This is in line with previous reports [12]. As in other series, the majority of pancreatic head insulinomas were not approached minimally invasive, especially if they were located deeper in the parenchyma [17]. Central pancreatectomy is also a viable option for parenchyma-sparing resection of localized insulinoma in selected patients with tumors located in the pancreatic body, which are not suitable for enucleation. In the analyzed patient, collective tumor localization and criteria for possible enucleation, however, did not warrant this type of approach. The comparable rates of clinically relevant complications observed between minimally invasive and open operations were to be expected, as POPF was the main reason for relevant complications in both groups (11% and 13%), which is independent from the type of surgical approach. This complication rate is in the range of other series [18]. In the present study, the most frequently performed operation for benign insulinoma in both time periods was parenchyma-sparing enucleation with 56% and 52%. A retrospective analysis of 80 insulinoma resections between 1989 and 2019 by de Carbonnières et al. reported a similar rate of parenchyma-sparing resections [19]. However, in other series, the rate of distal resections or pancreatectomies is much higher for the resection of small pNENs, including insulinoma [18].

Even though recurrence was detected in two patients during long-term follow-up, all benign tumors were surgically cured. This confirms the reports of other studies with long-term follow-up [18, 19]. Therefore, long-term follow-up investigations after R0 resection of benign sporadic insulinoma appear to be only necessary in case of recurrent symptoms. This is in line with current ENETS recommendations, where a single follow-up 3–6 months postoperatively with CgA measurement (if initially elevated) and *no* recurrent imaging is proposed for the specific pNEN subgroup “Insulinoma, solitary, G1–G2 NET” [20].

All 8 patients with MEN1-associated benign insulinoma in the present cohort were also surgically cured of their organic hyperinsulinism, which supports recommendations for a potentially parenchyma-sparing approach for this subgroup as well [11]. However, the underlying MEN1 syndrome requires a lifelong, systematic screening for all possible tumor manifestations [11]. Although multiple insulinomas in MEN1 patients are reported in the literature, we did not yet observe this condition in our MEN1 cohort of over 100 patients. All MEN1 patients with organic hyperinsulinism in our collective were biochemically cured after surgery and none of these patients developed a relapse during long-term follow-up so far.

The very rare cases of malignant insulinoma presented quite heterogenous and adjustments in treatment ranged from slightly more radical surgical approaches (lymphadenectomy, formal resections) in intermediately aggressive tumors to a even non-curable, palliative situation in our patient with a G3 insulinoma. These rare patients require a tailored multimodal approach including PRRT, transarterial chemoembolization (TACE), chemotherapy, and/or targeted therapy e.g. with everolimus as in our patient, focusing primarily on treatment of the severe hypoglycemia [21]. A recent retrospective multicenter series of 31 patients with malignant insulinoma collected over 30 years from Italy showed that 35% of these patients died during follow-up (median follow-up 60 months, range 3–194 months). Ten of 31 patients received no surgical intervention, whereas all others received resection alone or a combined surgical and systemic approach upfront. Correlations with a better overall survival in these 31 patients were found for Ki67 ≤ 10% (*P* = 0.03), an insulin level < 60 μU/mL at diagnosis (*P* = 0.015), and in patients who underwent surgery (*P* = 0.006) [22].

Our study has its main strengths in a rather large cohort of insulinoma patients with a long-term follow-up. Almost all diagnostic data was generated and documented in-house in the same institution as the surgical procedures and outcomes. The main weakness of this study is its monocentric, retrospective, purely descriptive concept. Even though the data were acquired prospectively in the years 2000 until 2020, there are still inherent shortcomings like individual missing data points, for example in older pathological reports (i.e., regarding proliferation rate and resection margins).

## Conclusion

Nowadays, insulinoma can be preoperatively localized in almost all patients, allowing for a minimally invasive, parenchyma-sparing resection in selected cases. The long-term cure rate of benign insulinoma after parenchyma-sparing surgery is excellent. Endoscopic ultrasound, if performed by an expert, appears to be the most effective tool for the preoperative detection of insulinoma.

## Data Availability

The data that support the findings of this study are not openly available due to reasons of sensitivity and are available from the corresponding author upon reasonable request.

## Declarations

This retrospective study was performed in adherence to local ethical standards and guidelines.
